# Editorial: Recent advances in understanding sex-based tumor diversity

**DOI:** 10.3389/fphar.2025.1598740

**Published:** 2025-04-28

**Authors:** Swati Kushwaha, Radha Kushwaha, Prem Prakash Kushwaha

**Affiliations:** ^1^ Department of Botany, University of Allahabad, Allahabad, Uttar Pradesh, India; ^2^ Centre of Food Technology, University of Allahabad, Allahabad, Uttar Pradesh, India; ^3^ Department of Biological, Geological, and Environmental Sciences, Cleveland State University, Cleveland, OH, United States

**Keywords:** Sex specific tumor heterogeneity, cancer biology, tumor progression, precision oncology, therapeutic targets

## Introduction

Sex differences play a fundamental role in cancer incidence, progression, and treatment response; however, their influence remains underexplored in many aspects of oncology. Emerging research suggests that biological factors such as sex hormones, genetic variations, immune system differences, and metabolic pathways contribute to distinct tumor behavior and therapeutic outcomes between men and women. Understanding these differences is critical to advancing precision medicine and improving patient care. This Research Topic, *Recent advances in understanding sex-based tumor diversity*, brings together 10 cutting-edge studies that investigate the impact of sex on cancer biology across a range of malignancies. These investigations examine molecular mechanisms, prognostic markers, and therapeutic targets, revealing how sex affects tumor initiation, progression, immune responses, and treatment resistance, paving the way for personalized therapies.

## Article description

The first review, published by Zhu and Zhao, explored the influence of sex on bladder cancer, from incidence to biology and outcomes. They assessed risk factors such as smoking, occupational exposures, and genetic mutations, along with sex differences in cancer development. They provided comprehensive insights into the role of hormones, chromosomes, metabolism, and the microbiome, while also highlighting gender gaps in diagnosis and prognosis. Their work underscores the need for further research and sex-specific treatments to improve care for all patients.

Li et al. analyzed cutaneous melanoma using TCGA and GEO data, integrating mutational, clinical, and single-cell sequencing to identify key genes. They developed a prognostic model using LASSO, linking genes to immune responses through functional and immunological analyses. They demonstrated the role of GMR6 in melanoma cell proliferation, invasion, and migration. These findings identify potential therapeutic targets, offering new avenues to improve patient outcomes in cutaneous melanoma, particularly by targeting GMR6-mediated mechanisms.

A mini-review (Su et al.) explored sex differences in hepatocellular carcinoma, focusing on sex hormones, genetics, and environmental factors. The authors highlighted the sexual dimorphism of the liver and sex-specific risks such as alcohol and obesity in cancer development. The review explores molecular mechanisms, including androgen and estrogen signaling, that influence tumor biology and highlights the need for sex-specific research to improve diagnostics, treatment precision, and personalized therapies, aiming to enhance outcomes for liver cancer patients.

Wu et al. investigated the role of AIB1 in endometrial cancer, focusing on its impact on aerobic glycolysis and tumor progression. AIB1 is linked to poor prognosis and has been identified as a potential therapeutic target. Using cell lines and mouse models, researchers confirmed the high expression of AIB1 and its role in tumor proliferation and invasion. Mechanistically, AIB1 is acetylated by PCAF, binds to c-myc, and regulates glycolysis-related genes. These findings suggest that targeting AIB1-mediated glycolysis may offer a novel strategy for the treatment of estrogen-dependent endometrial cancer, thereby advancing personalized therapies.

Another study (Xia et al.) investigated the chromatin accessibility in peripheral blood mononuclear cells (PBMCs) from breast cancer patients to assess its diagnostic and prognostic potential. Using ATAC sequencing and bioinformatic analysis, they identified 1,906 differentially accessible regions and 1,632 differentially expressed genes. Nine key genes (e.g., JUN, CDC42, TRIB1) and five transcription factors (NFY, Sp2, ELK1) were linked to breast cancer progression. Their findings suggest that chromatin accessibility in PBMCs is a promising biomarker for early detection and therapeutic innovation in breast cancer.

Yu et al. investigated BYL-719, a PI3K p110α inhibitor, to target breast cancer stem cells (BCSCs) and overcome drug resistance. Using a 3D mammosphere model, they found that BYL-719 inhibits BCSC proliferation, stemness, and epithelial-to-mesenchymal transition (EMT). The drug suppresses key pathways such as PI3K/AKT/mTOR, Notch, JAK-STAT, and MAPK/ERK that regulate the dynamics of the tumor microenvironment. The authors demonstrated that BYL-719 overcomes resistance in eribulin-resistant breast cancer cells. These results suggest that combining BYL-719 with other therapies may enhance breast cancer treatment strategies.

Yu et al. explored cell communication in hepatocellular carcinoma (HCC) using single-cell sequencing and clustering analysis. They identified malignant and cancer-associated fibroblast subpopulations, emphasizing SPP1-mediated interactions. Two clusters, C1 and C2, were distinguished, with C1 showing higher cytotoxicity and invasion. A gene risk model revealed increased immune pathway activity in C1, while high-risk scores correlated with poorer prognosis. They showed that ABCA1 promotes HCC progression by enhancing proliferation, invasion, and migration while reducing apoptosis. These findings provide critical insights into the pathogenesis and prognosis of HCC.

Hong et al. investigated WNT signaling genes in melanoma, identifying 19 prognostically relevant genes and developing a 13-gene model using LASSO regression. Key findings linked CSNK1E and RAC3 to epithelial-to-mesenchymal transition and immune evasion. They identified a role for CSNK1E in melanoma progression via the TGF-β pathway. These results suggest that targeting CSNK1E and WNT/TGF-β pathways could improve melanoma treatment and address therapy resistance.

Wang et al. investigated inflammatory gene expression in epithelial ovarian cancer and its role in immunotherapy resistance. Transcriptome analysis revealed two inflammatory gene patterns that differed in immune infiltration, prognosis, and treatment response. The high-risk group showed elevated M2 macrophage infiltration, increased tumor stemness, poorer prognosis, and reduced sensitivity to chemotherapy and immune checkpoint inhibitors. These findings underscore inflammation-related genes as potential targets for enhancing immunotherapy and prognostic evaluation, offering new strategies for early intervention and patient management.

Zhang and Pang studied cell senescence-associated genes in thyroid cancer, developing a prognostic model using differential expression, Cox regression, and LASSO analyses. Validated with Kaplan-Meier and ROC curves, the model predicts patient survival, tumor mutation burden, and response to immunotherapy across risk groups. Their findings offer new insights into thyroid cancer progression and immunotherapy, highlighting potential avenues for personalized treatment strategies.

Collectively, these studies advance our understanding of cancer biology by uncovering sex differences, molecular mechanisms, and therapeutic targets, paving the way for personalized treatments and improved patient outcomes across multiple malignancies.

